# Instillation of Sericin Enhances Corneal Wound Healing through the ERK Pathway in Rat Debrided Corneal Epithelium

**DOI:** 10.3390/ijms19041123

**Published:** 2018-04-09

**Authors:** Noriaki Nagai, Yuya Fukuoka, Miyu Ishii, Hiroko Otake, Tetsushi Yamamoto, Atsushi Taga, Norio Okamoto, Yoshikazu Shimomura

**Affiliations:** 1 Faculty of Pharmacy, Kindai University, 3-4-1 Kowakae, Higashi-Osaka, Osaka 577-8502, Japan; 1833420015f@kindai.ac.jp (Y.F.); 1833420012r@kindai.ac.jp (M.I.); hotake@phar.kindai.ac.jp (H.O.); yamatetsu@phar.kindai.ac.jp (T.Y.); punk@phar.kindai.ac.jp (A.T.); 2 Department of Ophthalmology, Kindai University Faculty of Medicine, 377-2 Ohno-Higashi, Osaka-Sayama, Osaka 589-8511, Japan; eyedoctor9@msn.com (N.O.); yoshis@med.kindai.ac.jp (Y.S.)

**Keywords:** sericin, cornea, cell proliferation, wound healing, MAPK/ERK pathway

## Abstract

Sericin is a major constituent of silk produced by silkworms. We previously found that the instillation of sericin enhanced the proliferation of corneal epithelial cells, and acted to promote corneal wound healing in both normal and diabetic model rats. However, the mechanisms by which sericin promotes the proliferation of corneal cells have not been established. In this study, we investigated the effects of sericin on Akt and ERK activation in a human corneal epithelial cell line (HCE-T cells) and rat debrided corneal epithelium. Although Akt phosphorylation was not detected following the treatment of HCE-T cells with sericin, ERK1/2 phosphorylation was enhanced. The growth of HCE-T cells treated with sericin was significantly increased, with the cell growth of sericin-treated HCE-T cells being 1.7-fold higher in comparison with vehicle-treated HCE-T cells. On the other hand, both of an ERK inhibitor U0126 (non-specific specific inhibitor) and SCH772984 (specific inhibitor) attenuated the enhanced cell growth by sericin, and the growth level in the case of co-treatment with sericin and ERK1/2 inhibitor was similar to that of cells treated with ERK1/2 inhibitor alone. In an in vivo study using rat debrided corneal epithelium, the corneal wound healing rate was enhanced by the instillation of sericin, and this enhancement was also attenuated by the instillation of U0126. In addition, the corneal wound healing rate in rats co-instilled with sericin and U0126 was similar to that following the instillation of U0126 alone. In conclusion, we found that the instillation of sericin enhanced cell proliferation via the activation of the MAPK/ERK pathway, resulting in the promotion of corneal wound healing in rat eyes. These findings provide significant information for designing further studies to develop potent corneal wound-healing drugs.

## 1. Introduction

The cornea is a protective barrier and the primary refractive element of the visual system. The cornea consists of five parts: the corneal epithelium, the Bowman layer, the corneal stroma, the Descemet membrane, and the corneal endothelium, with the corneal epithelium being the outermost layer of the cornea. The corneal epithelium is generated by limbal stem cells in the corneoscleral junction [1]. This mechanism maintains the transparency and integrity of the cornea. On the other hand, the corneal epithelium can suffer damage from the external environment, and this damage can result in scarring or opacification, resulting in visual defects that compromise transparency and that can even lead to a complete loss of vision. A wound in the corneal epithelium initiates multiple complex cellular processes [2]. The repair of the damaged corneal epithelium involves the frequent movement of cells at the wound edge; after that, cell proliferation is the leading process. Eventually, basement membrane and restoration of the stratification is achieved [3]. The increase in the centripetal movement and cell proliferation of corneal epithelial cells acts to promote corneal wound healing. In addition, many of these biological processes are also mediated by growth factors, cytokines and other mediators released by injured tissues or cells [4]. In particular, cell proliferation via growth factors has been recognized as an important mediator of proper wound repair [5]. Therefore, an increase in the proliferation of corneal epithelial cells may act to promote corneal wound healing.

Sericin is a major constituent of silk produced by silkworms [6], and has long been used for many purposes in the biomedical field. The sericin is a potent natural antioxidant due to its high content of hydroxyl amino acids [7], and it has been reported that sericin acts to promote anticoagulation activity [8], antagonizing oxidation stress [9] and cell proliferation [10]. Moreover, an increasing number of studies have demonstrated that the sericin protein is not immunogenic [11,12,13], and promotes wound healing and collagen deposition by fibroblasts and keratinocytes [14]. We have also previously reported that the instillation of sericin enhances the proliferation of corneal epithelial cells, and acts to promote corneal wound healing in both normal and diabetic model rats [15,16,17]. Therefore, sericin may be effective for the treatment of corneal wounds. However, the mechanism by which sericin promotes cell proliferation is not clear, and it is important to clarify the pathway of cell proliferation by corneal epithelial cells to develop safe and effective corneal wound-healing drugs.

It is well known that the PI3K/Akt/mTOR and MAPK/ERK pathways are mainly signaling for cell proliferation [18,19,20,21,22]. In this study, we investigated whether the PI3K/Akt/mTOR and MAPK/ERK pathways are related to the enhancement of corneal wound healing by the instillation of sericin.

## 2. Results

### 2.1. Effect of Sericin on the Akt and ERK Signalings in HCE-T Cells

**Figure 1:**
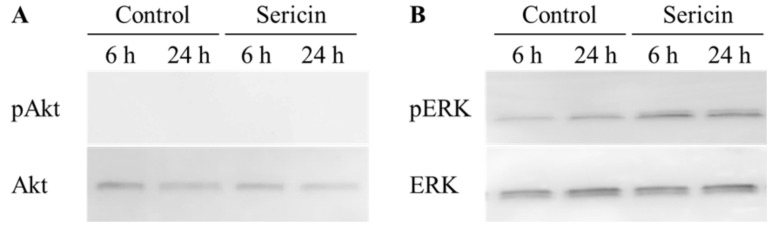
Changes in Akt (**A**) and ERK1/2 (**B**) activation in human cornea epithelial cell line (HCE-T) cells treated with 0.01% sericin. Sericin was dissolved in saline. Samples were obtained 6 and 24 h after the addition of sericin. Sericin treatment enhanced the phosphorylation of ERK1/2.

**Figure 2:**
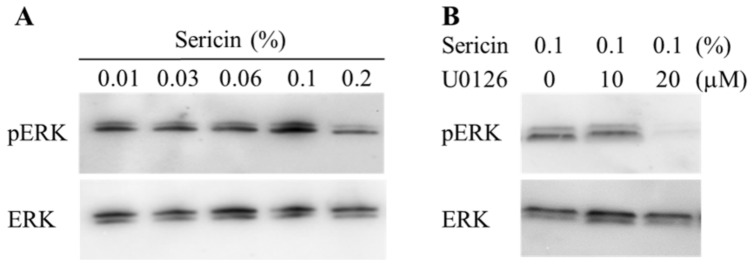
Effect of 0.01–0.2% sericin (**A**) and 0–20 μM U0126 (**B**) on the phosphorylation of ERK1/2 in HCE-T cells. (**A**) 0.01–0.2% sericin was dissolved in 0.05% DMSO, and treated for 24 h. (**B**) 0–20 μM U0126 was dissolved in 0.05% DMSO, and treated for 24 h. The phosphorylation of ERK1/2 was enhanced with the increase in sericin concentration; 20 μM U0126 prevented the phosphorylation of ERK1/2 in HCE-T cells.

**Figure 3:**
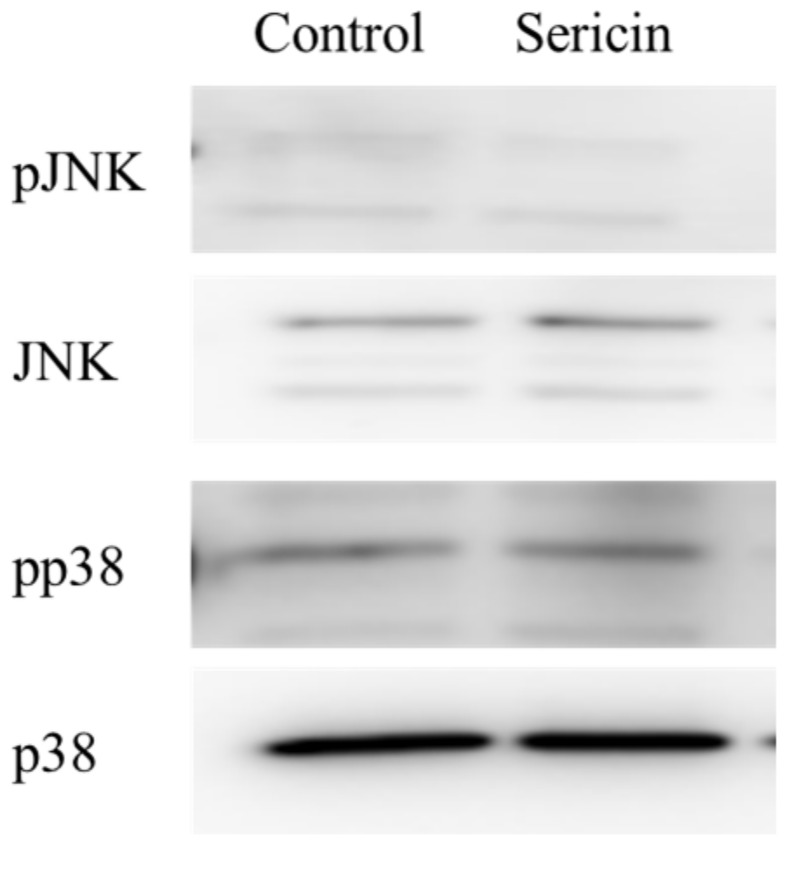
Changes in JNK and p38 activation in HCE-T cells treated with 0.1% sericin. Sericin was dissolved in saline. Samples were obtained 24 h after the addition of saline (control) or sericin. Sericin treatment did not affect the phosphorylation of JNK and p38.

**Figure 4:**
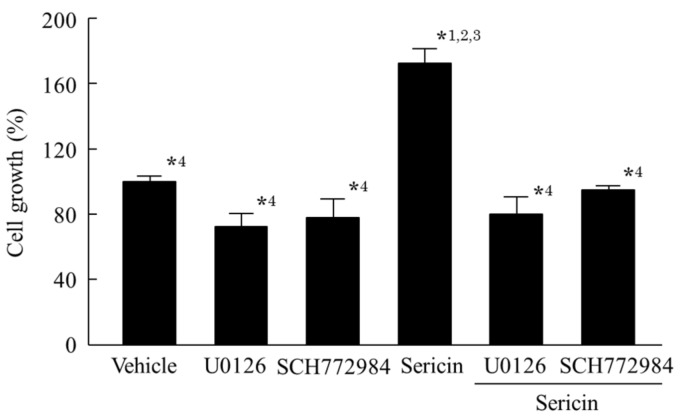
Effect of U0126 on the proliferation of HCE-T cells treated with sericin. Twenty micromoles of U0126, 0.5 μM SCH772984, and/or 0.1% sericin were dissolved in 0.05% DMSO, and treated for 24 h. Vehicle, 0.05% DMSO-treated HCE-T cells. U0126, U0126-treated HCE-T cells, SCH772984, SCH772984-treated HCE-T cells. Sericin, sericin-treated HCE-T cells. Sericin + U0126, sericin and U0126 co-treated treated HCE-T cells. Sericin + SCH772984, sericin and SCH772984 co-treated HCE-T cells. *n* = 7. *^1^ *p* < 0.05, vs. vehicle for each category. *^2^ *p* < 0.05, vs. U0126 for each category. *^3^ *p* < 0.05, vs. SCH772984 for each category. *^4^ *p* < 0.05, vs. sericin for each category. The enhanced growth in HCE-T cells treated with sericin was prevented by the addition of U126 and SCH772984.

Figure 1 shows the phosphorylation of the Akt and ERK1/2 proteins in a human corneal epithelial cell line (HCE-T cells) treated with 0.01% sericin. The phosphorylation of Akt was not detected in this study. In contrast to the results with Akt, the phosphorylation of ERK1/2 was enhanced by treatment with sericin. Figure 2A shows the changes in the phosphorylation of the ERK1/2 protein in HCE-T cells treated with 0.01–0.2% sericin. The phosphorylation of ERK1/2 increased with sericin concentrations in the range of 0.01–0.1%. On the other hand, ERK1/2 phosphorylation in HCE-T cells treated with 0.2% sericin was lower than in cells treated with 0.1% sericin. Figure 2B shows the effect of U0126 on the phosphorylation of the ERK1/2 protein in 0.1% sericin-treated HCE-T cells. Ten micromolar U0126 had no effect on the phosphorylation of ERK1/2; however, no band corresponding to the phosphorylation of ERK1/2 was detected in 0.1% sericin-treated HCE-T cells following treatment with 20 μM U0126. Figure 3 shows the phosphorylation of the c-jun N-terminal kinase (JNK) and p38 proteins in the HCE-T cells treated with 0.01% sericin. The phosphorylation of JNK and p38 were similar in HCE-T cells with or without treatment of sericin. Figure 4 shows changes in the growth of HCE-T cells following co-treatment with 0.1% sericin and ERK inhibitor (20 μM U0126 and 0.5 μM SCH772984). The growth of HCE-T cells treated with sericin was significantly increased, and the growth levels of sericin-treated HCE-T cells was 1.7-fold higher in comparison with vehicle-treated HCE-T cells. On the other hand, treatment with U0126 inhibited the enhanced cell growth by sericin, so that the growth levels of HCE-T cells co-treated with sericin and U0126 were similar to those of cells treated with U0126 alone. In addition, the SCH772984 also prevented the enhanced cell growth by the sericin.

### 2.2. Enhancement of the Corneal Wound Healing by Sericin via ERK1/2

**Figure 5:**
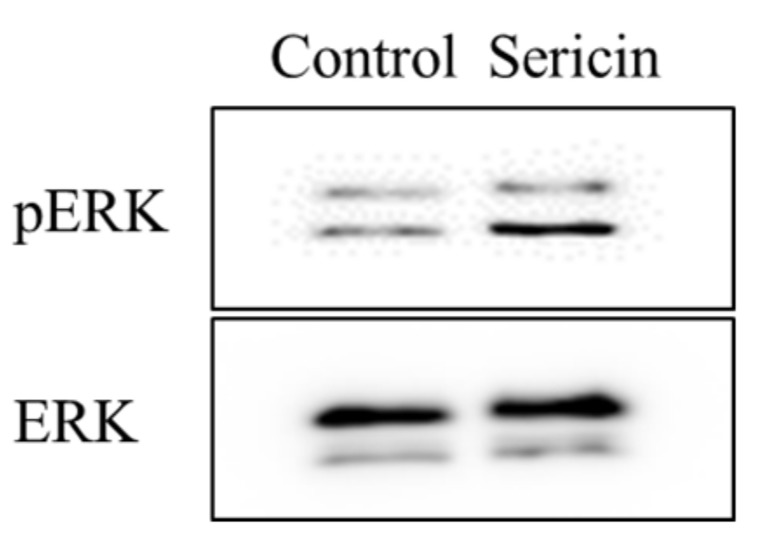
Effect of sericin on ERK1/2 activation in rat debrided corneal epithelium. One percent sericin was dissolved in saline, and the saline (control) or sericin was instilled into the eyes of rats five times a day. The cornea was collected 24 h after corneal epithelial abrasion. The instillation of sericin enhanced the phosphorylation of ERK1/2 in the cornea of rats.

**Figure 6:**
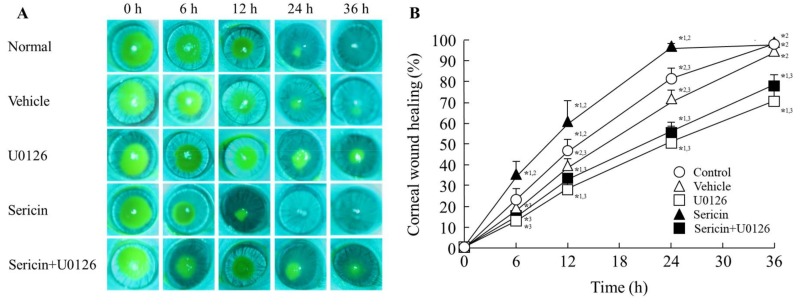
Corneal image (**A**) and corneal wound healing (**B**) in rat eyes treated with sericin and/or U0126. 1% sericin and 200 μM U0126 were dissolved in 0.2% DMSO, and instilled into the eyes of rats five times a day. The corneal images were obtained by a TRC-500X, and corneal wound healing was calculated according to Equation (2). Normal (○), non-instilled rat. vehicle (△), 0.2% DMSO-instilled rat. U0126 (□), U0126-instilled rat, sericin (▲), sericin-instilled rat. Sericin + U0126 (■), sericin-instilled rat treated with U0126. *n* = 5–8. *^1^ *p* < 0.05, vs. vehicle for each category. *^2^ *p* < 0.05, vs. U0126 for each category. *^3^ *p* < 0.05, vs. sericin for each category. The corneal wound healing was increased by the instillation of sericin, and the enhanced corneal wound healing in rats instilled with sericin was prevented by treatment with U0126.

**Figure 7:**
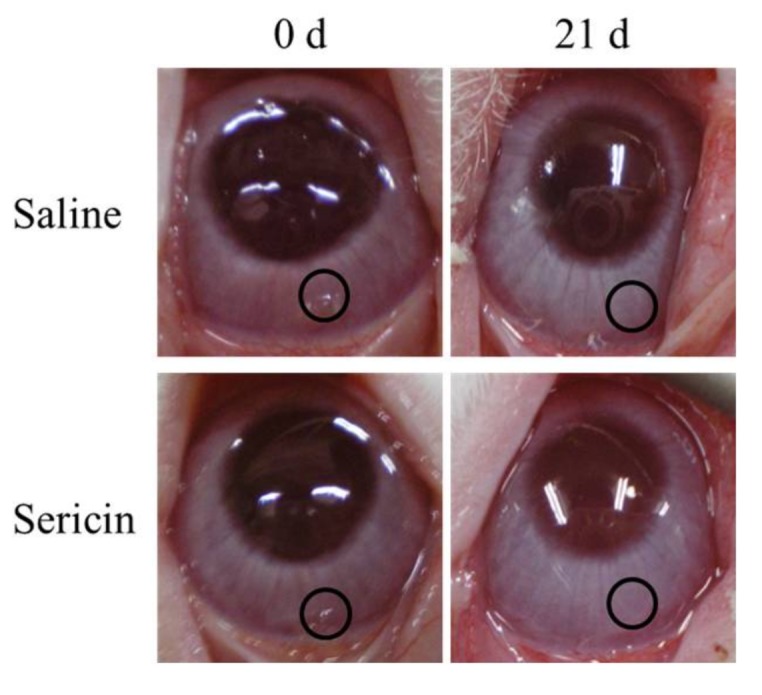
Effects of continuous treatment with sericin on eyes in rabbits. The sustained-release hydrogel containing saline and/or sericin was injected into the conjunctiva (black circles) and maintained for 21 days. No abnormal changes, such as angiogenesis, were observed in the rabbit eyes 21 days after insertion of hydrogel containing sericin.

Figure 5 shows the phosphorylation of ERK1/2 in the cornea of rat 24 h after corneal abrasion. The phosphorylation of ERK1/2 was increased by the instillation of sericin. Figure 6 shows images of rat eyes following corneal epithelial abrasion as documented by a TRC-50X (Figure 6A), and corneal wound healing of the eyes following the instillation of 1% sericin and/or 200 μM U0126 (Figure 6B). Table 1 shows the constant of corneal wound healing rates (*k*_H_) in rats instilled with sericin and/or U0126. The healing of corneal wounds in non-instilled rats was approximately 45% 12 h after abrasion, and the corneal wounds were almost entirely healed 36 h after abrasion. The corneal wound healing rate was enhanced by the instillation of sericin, with a *k*_H_ value 1.35-fold higher than in non-instilled rats. On the other hand, the instillation of U0126 decreased the rate of corneal wound healing, and U0126 also attenuated the enhancement of *k*_H_ by the instillation of sericin. The *k*_H_ value in rats co-instilled with sericin and U0126 was similar to that in rats instilled with U0126 alone. Figure 7 shows the changes in eyes of rabbits by the continuous treatment with sericin. There were no differences observed between eyes treated with saline or sericin, and no angiogenesis was observed in rabbit eyes 21 days after the insertion of hydrogel containing sericin.

## 3. Discussion

Sericin has been shown to have mitogenic activity and to prevent cell death from several stimuli, including hyperthermia, and we also reported that the instillation of sericin increased cell proliferation, resulting in an enhancement in the corneal wound healing rate of rats with or without diabetes mellitus [15,16,17]. However, the mechanisms by which sericin promotes the proliferation of corneal cells have not been established. In the present study, we investigated the molecular mechanisms for the effect of sericin on the corneal wound healing process, and found that the sericin enhanced cell proliferation via the activation of ERK1/2.

The PI3K/Akt/mTOR and MAPK/ERK pathways are the major pathways to increase cell proliferation, and it has been reported that the loss of Akt activity leads to cellular dysfunction and delayed corneal wound healing [23]. In addition, Hong et al. [24] reported that the nerve growth factor (NGF) lead expression of D-type cyclin, and the enhanced D-type cyclin shortens the cell cycle by activating Akt and ERK signaling, resulting in enhancement of the proliferation in corneal epithelial cells. Moreover, the activation of LY294002 (Akt inhibitor) or PD98059 (ERK inhibitor) can significantly prevent the Akt and ERK via the inhibition of D-type cyclin. Based on these previous studies, we demonstrated the effect of sericin on Akt and ERK signaling in cultured cells (corneal epithelial cells, HCE-T cells). The phosphorylation of Akt in the HCE-T cells was not detected in the normal condition, and the Akt phosphorylation was also not detected following treatment with sericin (Figure 1). Therefore, the PI3K-Akt-mTOR pathway may be not have a lot of involvement in the HCE-T cells. In contrast to the results with Akt, sericin treatment was found to enhance ERK1/2 phosphorylation (Figure 1). Our previous in vitro study using HCE-T cells showed that the cell growth activity was increased by treatment with 0.01–0.5% sericin solution, reaching a maximum at 0.1% sericin, and subsequently decreasing [16]. In this study, the phosphorylation of ERK1/2 was increased at sericin concentrations of 0.01–0.1%, and the phosphorylation of ERK1/2 in HCE-T cells treated with 0.2% sericin was lower than that in cells treated with 0.1% sericin (Figure 2A). Furthermore, the enhanced phosphorylation of ERK1/2 in 0.1% sericin-treated HCE-T cells was attenuated by co-treatment with 20 μM U0126 (Figure 2B), and the proliferation of cells co-treated with sericin and U0126 was similar to that of cells treated with U0126 alone (Figure 4). In addition, the SCH772984 also prevented the proliferation of cells treated with sericin (Figure 4), and phosphorylation of JNK and p38 were not led by the sericin (Figure 3). These results suggested that sericin caused the enhancement of cell proliferation via activation of the MAPK/ERK pathway. On the other hand, it cannot be elucidated sufficiently about the difference of ERK1/2 phosphorylation between 0.1% and 0.2% sericin treatment. We do not have data to clarify the mechanism for the decrease in pERK1/2 levels after using 0.2% sericin, however, it was reported that some factor, such as dual-specificity phosphatase, is able to interact with ERK [25]. The high concentration of sericin may result in non-specific binding, and the 0.2% sericin may affect the same factor, resulting in the prevention of ERK1/2 phosphorylation. Further consideration is needed.

Next, we investigated whether ERK signaling was related the enhancement of corneal wound healing by sericin in an in vivo study. The concentration of drugs administered as eye drops is reduced to approximately 20% by dilution in the lacrimal fluid, and the components of eye drops are excreted though the nasolacrimal duct into the mouth, and so have a short residence time in the eye [26]. Our previous reports also showed that the instillation of 1–10% sericin solution enhances corneal wound healing [16]. Therefore, we used high concentrations of sericin and ERK inhibitor (U0126) in the in vivo experiment, and the concentrations chosen were 10 times than used in the in vitro experiment using HCE-T cells. The instillation of sericin enhanced the rate of corneal wound healing via phosphorylation of ERK1/2 (Figure 5 and Figure 6), and U0126 attenuated the enhancement of *k*_H_ by sericin (Table 1). These results support the in vitro experiment in HCE-T cells (Figure 1, Figure 2, Figure 3 and Figure 4). On the other hand, judging from the presented graphs and eye photos, sericin accelerates the rate of wound healing, but the full healing occurs at about the same time as in controls. It is known that the corneal epithelial was maintained by the balance of cell proliferation (*X*), centripetal migration (*Y*), and desquamation (*Z*) (*X* + *Y* = *Z*) [27,28,29,30], and the corneal wound is healed by the following steps: epithelial cell loss from the corneal surface (*Z*) shrinks and covers the corneal wound surface (*Y*), after that the rebuilding and remodeling of tissue is provided by cell proliferation (*X*) [27,28,29,30]. Thus, the initial steps of corneal wound healing are cell migration with polarization and protrusion in the direction of migration [31], and the decrease in the wound area during the 12 h after corneal epithelial abrasion due to cell migration [32]. Moreover, the MAPK/ERK pathways participate in the regulation of the manifold pathological and physiological processes [33,34,35,36], and it is well known that MAPK family members, such as ERK1/2, are also important for cell migration [19,20,21,22]. In this study, sericin enhanced cell movement, since the corneal wound healing in rats instilled with sericin was greater 12 h after corneal epithelial abrasion than in saline instilled rats. This suggests that sericin leads to an enhancement of corneal cell migration after corneal injury. On the other hand, corneal wound healing in rats co-instilled with sericin and U0126 tends to be than in rats instilled with U0126 alone 12 h after corneal abrasion (Figure 6). Taken together, The ERK signaling may not affect the enhancement of cell movement by sericin. Cell migration is caused by cytokines and growth factors, and requires the integration of key events in the signaling, adhesion processes, and cytoskeletal reorganization [37]. Therefore, cytokines and growth factors outside the ERK pathway may be related to the enhancement of corneal cell migration by sericin. Further investigation of the mechanism of cell movement by sericin is required in the future.

The cornea is avascular under normal conditions. Previous reports indicate that corneal avascularity was maintained by the production of antiangiogenic factors after corneal injury, low levels of proangiogenic matrix metalloproteinases, the immune privilege of the cornea by transforming growth factor-β in tears, and the angiostatic nature of corneal epithelial cells [38,39]. The persistent avascularity of the cornea is important for vision clarity. Therefore, we investigated changes in the corneal condition during the active production of factors via MAPK/ERK signaling by sericin. Angiogenesis was not observed in rabbit eyes treated with sericin, and no differences were apparent between rabbit eyes treated with saline and those treated with sericin (Figure 7). From these results, the instillation of sericin may provide an effective and safe corneal wound-healing drug.

Further studies are needed to clarify the precise mechanism for cell movement (cell–cell adhesion) following the instillation of sericin. In addition, it is also important to elucidate the mechanism of corneal wound healing by sericin in diabetic keratophathy. Therefore, we next plan to investigate the effects of sericin on corneal wound healing in Otsuka Long-Evans Tokushima Fatty rat, which is model of diabetic keratopathy [16,17].

## 4. Materials and Methods

### 4.1. Materials

Pure Sericin™ (30 kDa), U0126 and MedGel^®^ were obtained from Wako Pure Chemical Industries (Osaka, Japan), SCH772984 was purchased from Cayman Chemical (Ann Arbor, MI, USA). The disposable dermatological skin punch (BIOPSY PUNCH) and a BD Micro-Sharp™ (blade 3.5 mm, 30°) were provided by Kai Industries Co., Ltd. (Gifu, Japan) and Becton Dickinson (Fukushima, Japan), respectively. Dulbecco’s modified Eagle’s medium/Ham’s F12, 1000 IU/mL penicillin, 0.1 mg/mL streptomycin and heat-inactivated fetal bovine serum were purchased from GIBCO (Tokyo, Japan). A cell scraper was from ASAHI GLASS Co. Ltd. (Tokyo, Japan), and Cell Count Reagent SF was provided by Nacalai Tesque (Kyoto, Japan). Phospho-Akt(Ser473)(587F11) mouse mAb, Phospho-p44/42MAPK (Erk1/2)(Thr202/Tyr204)(D13.14.4E) XP^®^Rabbit mAb, Akt(pan)(40D4) mouse mAb, and P44/42MAPK(Erk1/2) antibody were obtained from Cell Signaling Technology, Inc. (Danvers, MA, USA). Affinity purified secondary antibodies against rabbit or mouse IgG (H and L) adsorbed against rat, human, bovine, and horse and conjugated to Horseradish Peroxidase (secondary anti-mouse or -rabbit IgG) were from American Qualex (San Clemente, CA, USA). One percent fluorescein and 0.4% Benoxil were purchased from Alcon Japan (Tokyo, Japan) and Santen Pharmaceutical Co., Ltd. (Osaka, Japan), respectively. All other chemicals were of the highest purity commercially available.

### 4.2. Animals

Seven-week-old male Wistar rats and Japanese albino rabbits (2.5 kg) were purchased from Kiwa Laboratory Animals Co., Ltd. (Wakayama, Japan) and Shimizu Laboratory Supplies Co. Ltd. (Kyoto, Japan), respectively. All experiments were performed in accordance with the ARVO resolution on the use of animals in research, and were approved by the Kindai University Faculty of Pharmacy Committee Guidelines for the Care of Laboratory Animals (project identification code KAPS-27-017, 1 April 2015).

### 4.3. Cell Culture

The immortalized human cornea epithelial cell line (HCE-T) developed by Araki-Sasaki et al. [40]. The HCE-T cells were cultured under humidified air containing 5% CO_2_ at 37 °C in Dulbecco’s modified Eagle’s medium/Ham’s F12 containing 1000 IU/mL penicillin, 0.1 mg/mL streptomycin and 5% (*v*/*v*) heat-inactivated fetal bovine serum. Sericin, U0126 (MAPK/ERK inhibitor, U0126 inhibits activation of ERK1/2) and SCH772984 (ERK1/2 selective inhibitor) were added to the cell cultures 1 day after seeding (1 × 10^4^ cells) by changing the culture medium, and then incubated for 6 and 24 h in 96-well microplates (IWAKI, Chiba, Japan). After that, Cell Count Reagent SF was added, and the absorbance (Abs) at 450 nm was measured according to the manufacturer’s protocol. Cell proliferation is represented by the following Equation (1) [16]:

Cell growth (%) = Abs_treatment_/Abs_non-treatment_ × 100 (1)

in the study using cultured cells, sericin, U0126 and SCH772984 were dissolved in saline and 0.05% DMSO, respectively.

### 4.4. Western Blot Analysis

HCE-T cells were cultured in 25 cm^2^ culture flasks. During cell collection, the growth medium was removed, and the cells were rinsed twice with 3 mL phosphate buffered saline prior to lysis with radioimmunoprecipitation assay (RIPA) buffer. After that, the cell lysates were collected with a cell scraper, and centrifuged at 17,900× *g* for 10 min at 4 °C. The supernatants were boiled for 3 min, and used the measurement of Western blot analysis. In the measurement of protein in rat cornea, the rat cornea was collected 24 h after corneal epithelial abrasion, and homogenized in urea lysis buffer (7 M urea, 2 M thiourea, 5% CHAPS, and 1% Triton X-100). Total protein (10 μg) of these samples (HCE-T cells and rat cornea) was separated in a 10% polyacrylamide SDS gel. The proteins were then transferred to polyvinylidene difluoride membranes (Bio-Rad, Hercules, CA, USA) using a semi-dry transfer cell (Trans-Blot SD Semi-Dry Electrophoretic Transfer Cell, Bio-Rad, CA, USA). The phosphorylation of Akt (pAkt), ERK1/2 (pERK), JNK (pJNK) and p38 (pp38) were probed with phospho-Akt(Ser473)(587F11) mouse mAb (1:1000 dilution), phospho-p44/42MAPK (Erk1/2)(Thr202/Tyr204)(D13.14.4E) XP^®^ rabbit mAb (1:1000 dilution), Phospho-SAPK/JNK(Thr183/Tyr185)(81E11) rabbit mAb (1:1000 dilution) and Phospho-p38 MAPK(Thr180/Tyr182)(D3F9) XP^®^ rabbit mAb (1:1000 dilution) for 10 h at 4 °C, respectively. After washing with Tris-buffer containing TEIRON X-100, the membranes were incubated with secondary anti-mouse or -rabbit IgG (1:4000 dilution, Promega, WI, USA) or HRP-conjugated anti-rabbit IgG antibody (1:4000 dilution; American Qualex, San Clemente, CA, USA) for 1 h at room temperature, and treated with 1 mL Super Signal^®^ and bands were detected using IQuant 400 for pAKT and pERK. The membranes were treated with 1 mL SuperSignal West Dura Extended Duration substrate (Thermo Fisher Scientific, Inc., Waltham, MA, USA) and bands were detected by the myECL Imager system for pJNK and pp38. Next, the membranes were washed with stripping buffer for 1 h, and the sites on the membranes were blocked with 5% non-fat dry milk in Tris-buffer (20 mM Tris-HCl, and 500 mM NaCl, pH 7.5), and used to measure the expression of Akt, ERK, JNK, and p38 proteins. Akt, ERK, JNK, and p38 were probed with Akt(pan)(40D4) Mouse mAb, P44/42MAPK(Erk1/2) antibody (1:1000 dilution), SAPK/JNK antibody (1:1000 dilution) and p38 MAPK(D13E1)XP^®^Rabbit mAb (1:1000 dilution), respectively, for 10 h at 4 °C. Then, secondary anti-mouse or -rabbit IgG (1:4000 dilution,) were added, and incubated for 1 h at room temperature.

### 4.5. Analysis of Corneal Wound Healing

Rats were anesthetized with isoflurane and Benoxil, and a patch of corneal epithelium was removed with a disposable dermatological skin punch and BD Micro-Sharp™ (9:00). The removed areas (corneal wound areas) were as follows: control (non-instillation), 12.39 ± 0.68; vehicle, 11.78 ± 0.56; U0126, 12.35 ± 0.53; sericin, 12.96 ± 0.47; sericin + U0126, 11.94 ± 0.85 (mm^2^, *n* = 5–8). For the in vivo study, sericin (1%) and U0126 (200 μM) were dissolved in 0.2% DMSO, and were instilled into the right eyes of rats five times a day (9:00, 12:00, 15:00, 18:00 and 21:00). The area of the corneal wounds was stained with fluorescein, and monitored by a TRC-50X (Topcon, Tokyo, Japan) equipped with a digital camera. The wound areas were evaluated with Image J, and analyzed by the following Equations (2) and (3) [16]:

Corneal wound healing (%) = (wound area 0 h − wound area 12, 24 or 36 h)/wound area 0 h × 100
(2)

(3) $$H_{t}=H_{\infty }\cdot (1-e^{-k_{H}\cdot t})$$

where *k*_H_ is the corneal wound healing rate constant (h^−1^), *t* is time (0–36 h) after corneal abrasion, and *H*_t_ and *H*_∞_ are the percentages of corneal wound healing (%) at times *t* and ∞, respectively.

### 4.6. Safety Evaluation of Sericin in the Cornea

Rabbits were anesthetized with isoflurane and Benoxil, and a pocket in the corneal stroma was made using a Crescent bevel up (ALCON Japan Ltd., Tokyo, Japan). A hydrogel complex containing saline and sericin was prepared as follows: 1 mg MedGel^®^ was added to 10 μL of saline with or without 5% sericin, and the mixture was incubated for 10 h at 4 °C. The hydrogel complex containing saline and sericin was inserted into the pocket in the conjunctiva. The changes in the eye were observed using a digital camera 21 days after the insertion of the hydrogel complex containing saline and sericin.

### 4.7. Statistical Analysis

Bar charts and line plots are expressed as the mean ± S.E.M., and a *p*-value of <0.05 was considered significant in his study. In the statistical analysis method, unpaired Student’s *t*-test, Aspin-Welch’s *t*-test, or Dunnett’s multiple comparison was selected.

## 5. Conclusions

We found that the instillation of sericin enhanced cell proliferation via the phosphorylation of proteins in the MAPK/ERK pathway, resulting in the promotion of corneal wound healing. These findings provide significant information for designing further studies to develop potent corneal wound-healing drugs.

## Figures and Tables

**Table 1:** Corneal wound healing rate constant (*k*_H_) in rats with or without the instillation of sericin and/or U0126.

Treatment	*k*_H_ (×10^−2^/h)
Control	4.81 ± 0.80 *^2,3^
Vehicle	4.25 ± 0.81 *^2,3^
U0126	1.48 ± 0.54 *^1,3^
Sericin	6.49 ± 0.82 *^1,2^
Sericin + U0126	1.63 ± 0.68 *^1,3^

One percent sericin and 200 μM U0126 were dissolved in 0.2% DMSO, and the solutions were instilled into the eyes of rats five times a day. The *k*_H_ was calculated according to Equation (3). Control, non-instilled rat. vehicle, vehicle-instilled rat. U0126, U0126-instilled rat, sericin, sericin-instilled rat. Sericin + U0126, sericin-instilled rat treated with U0126. *n* = 5–8. *^1^ *p* < 0.05, vs. vehicle for each category. *^2^ *p* < 0.05, vs. U0126 for each category. *^3^ *p* < 0.05, vs. sericin for each category.

## Abbreviations

Abs	Absorbance
HCE-T cells	Human corneal epithelial cell line
NGF	Nerve growth factor
pp38	Phosphorylation of p38
pAkt	Phosphorylation of Akt
pERK	Phosphorylation of ERK
pJNK	Phosphorylation of JNK
U0126	ERK inhibitor
